# Emergent Anthropometric Indices in Differential Prediction of Prehypertension and Hypertension in Mexican Population: Results according to Age and Sex

**DOI:** 10.1155/2022/4522493

**Published:** 2022-07-07

**Authors:** Oscar Zaragoza-García, Ilse Adriana Gutiérrez-Pérez, Pedro Delgado-Floody, Isela Parra-Rojas, Daniel Jerez-Mayorga, Christian Campos-Jara, Iris Paola Guzmán-Guzmán

**Affiliations:** ^1^Faculty of Chemical-Biological Sciences, Universidad Autónoma de Guerrero, Chilpancingo 39087, Guerrero, Mexico; ^2^Department of Physical Education, Sport, and Recreation, Universidad de La Frontera, Temuco 4780000, Chile; ^3^Exercise and Rehabilitation Sciences Laboratory, School of Physical Therapy, Faculty of Rehabilitation Sciences, Universidad Andres Bello, Santiago 7591538, Chile; ^4^Department of Physical Education and Sports, Faculty of Sport Sciences, University of Granada, Granada 18011, Spain

## Abstract

**Background:**

Hypertension (HTN) is recognized as a significant public health problem in the world. The objective of this study is to evaluate emergent anthropometric indices as predictors of preHTN and HTN according to age and sex in a sample of Mexican adults.

**Methods:**

A cross-sectional study was conducted in 1,150 participants aged 18–80 years old. Anthropometric data and blood pressure measurements were analyzed. Comparisons between men and women were carried out by independent analysis. Cutoff points for each emergent anthropometric index were obtained using the values' upper second and third tertiles. Logistic regression models and receiver operating characteristics curve analyses were used to assess the association and the predictive value of several emergent anthropometric indices with the presence of preHTN and HTN.

**Results:**

The prevalence of preHTN and HTN was 29.74% and 14.35%, respectively. In a logistic regression analysis adjusted by age and sex, the body roundness index (BRI) (OR = 2.08, *p* < 0.001) and conicity index (CI) (OR = 1.37, *p*=0.044) were associated with preHTN, while CI (OR = 2.47, *p* < 0.001) and waist to height squared (W/Ht^2^) (OR = 2.19, *p* < 0.001) were associated with HTN. Furthermore, in both sexes, BRI was the main predictor of preHTN (AUC: 0.634 and 0.656, respectively). Particularly, according to sex and age range, the predictive emergent anthropometric indices in men were the body shape index (ABSI) and waist to height cubic (W/Ht^3^) (AUC = 0.777 and 0.771, respectively), whereas in women, the predictors were CI and ABSI (AUC = 0.737 and 0.729, respectively). In men ≤40 years old, central body fat indices were predictors of preHTN and HTN, but in men >40 years old, the predictor indices were W/Ht^3^ and W/Ht^2^. In women ≤40 years, the pulse mass index (PMI) was the best main predictor (AUC = 0.909) of HTN.

**Conclusion:**

CI, PMI, W/Ht^3^, W/Ht^2^, and ABSI could represent differential predictors of preHTN and HTN between men and women according to age range.

## 1. Introduction

Prehypertension (preHTN) and hypertension (HTN) are modifiable risk factors that could lead to mortality by coronary heart disease and ischemic stroke in populations of both sexes [1–4]. In a meta-analysis study, preHTN was related to the carotid intima-media thickness, a predictor of heart disease, stroke, and cardiovascular mortality [5]. Similarly, the presence of HTN is described as a risk factor most significantly correlated with strokes in the world population, as well as a related factor to the increased incidence of stroke in the young population [6].

In Mexico, the prevalence of preHTN reaches from 26.5% to 47.4% [2, 7], while the frequency of HTN is as high as 25.5% and 49.2% [8, 9]. Therefore, early detection of preHTN and HTN can help reduce morbidity and mortality providing timely treatment, management, and prevention of associated comorbidities. Due to the close relation between age, gender, adiposity, preHTN, and HTN [2, 10, 11], the search of anthropometric marker predictors of cardiovascular risk (CVR) such as HTN has revolutionized the field.

Traditional markers such as waist to height ratio (WHtR) have shown higher sensitivity than the body mass index (BMI) and waist circumference (WC) in the evaluation of the CVR [12]. However, emergent markers such as the body roundness index (BRI) have been suggested as an alternative for WHtR [13], because it possesses a predictive capacity for CVD [14, 15], principally for HTN [14, 16, 17]. In addition, the body adiposity index (BAI), abdominal volume index (AVI), body shape index (ABSI), or conicity index (CI) could have a predictive ability for evaluating HTN [16–19], while the ponderal index (PI) could do the same for preHTN [20]. The emergent anthropometric measurements are suggested to assess the risk of morbidity and mortality in the population given the fact that they are simple, inexpensive, and noninvasive tools. However, it is not yet fully clear which emergent anthropometric index may be associated with preHTN and with HTN in Mexican population and whether these vary according to sex and age. The aim of this study is to determine the emergent anthropometric indices as predictors of preHTN and HTN among men and women according to age range in a Mexican population.

## 2. Materials and Methods

### 2.1. Design and Study Population

This study was carried out in 2019 in a general population in the city of Chilpancingo in the state of Guerrero, located in southern Mexico. The study followed a cross-sectional design and was conducted on randomly selected subjects. A total of 1,150 participants (aged 18–80 years old) were included. The group was comprised of women (*n* = 852) and men (*n* = 298). Participants were invited to attend the evaluation sites that had been previously set up by the researchers in health care and educational centres, preferably between 7 and 9:30 a.m., following indications such as fasting and no exercise for at least 8 hours before the test. Eligibility criteria for the present analysis were to be aged between 18 and 80 years old and to live in Chilpancingo. Participants with musculoskeletal disorders or any other medical or physical condition that could make the clinical and anthropometric evaluation impossible were excluded from this study. All participants agreed to be a part of the study by giving their written consent following the considerations of the Declaration of Helsinki. The study was approved by the Research Ethics Committee of the Universidad Autónoma de Guerrero.

### 2.2. Data Collection and Anthropometric and Blood Pressure Measurements

Information on sociodemographic characteristics and lifestyle habits was obtained by a questionnaire. The body composition was evaluated while wearing light clothing and barefooted, using a bioelectrical impedance technique with HBF-514C (Tanita Corporation, OMRON, IL, USA), which allowed to assess weight, body mass index (BMI), body fat percentage, and visceral fat. Height was determined using a portable stadiometer (Seca 240, Hamburg, Germany). The BMI was classified according to the World Health Organization's criteria: normal weight (BMI = 18.5–24.9 kg/m^2^), overweight (BMI = 25–29.9 kg/m^2^), and obesity (BMI ≥30 kg/m^2^) [21]. Waist circumference (WC) was measured at the level of the umbilicus, with the subject standing up. Hip girth was measured at the maximum circumference of the buttocks. The circumference of the left wrist was also measured at the level of the ulna distal of the styloid apophysis process. The circumference of the left arm was measured by identifying the midpoint between the bone protrusion of the acromion and the olecranon, along the nondominant arm, with the elbow flexed at 90°. Once the middle point was identified, the arm was dropped naturally and the ribbon was placed horizontally around the indicated point. The body circumferences were measured twice using a measuring tape with an accuracy of ±0.1 cm (Seca 201, Hamburg, Germany). All measurements were made by trained health personnel.

The pulse and blood pressure were measured twice by trained technicians after a 5-minute seated rest, using the left arm of the participants. Two consecutive measurements were obtained at 5-minute intervals using a baumanometer (HEM-712C, OMRON, IL, USA).

### 2.3. Definitions

Indices such as BMI = weight (kg)/height (m)^2^, waist to hip ratio (WHR) = WC (cm)/hip (cm), and WHtR = WC (cm)/height (m) are considered traditional anthropometric indices. These were evaluated given that some of them are incorporated in the definition of emergent indices considered in this study, such as: the arm-waist index (AWI), hip-wrist index (HWrI), waist-wrist index (WWrI), waist to hip to height ratio (WHHR), waist to height square (W/Ht^2^), waist to height cubic (W/Ht^3^), height cubic to waist cubic (H^3^/W^3^), waist-corrected BMI (wBMI), ponderal index (PI), body roundness index (BRI), body adiposity index (BAI), a body shape index (ABSI), conicity index (CI), body fat distribution index (BFDI), abdominal volume index (AVI), and pulse mass index (PMI). The emergent anthropometric indices were derived using the following formulae:

- Arm-waist index (AWI, cm): WC (cm)/left arm circumference (cm)
- Hip-wrist index (HWrI, cm): hip circumference (cm)/left wrist circumference (cm) [22].
- Waist-wrist index (WWrI, cm): WC (cm)/left wrist circumference (cm) [22].
- Waist to hip to height ratio (WHHR, m^−1^): WHR/height (m) [23].
- Waist to height square (W/Ht^2^, cm/m^2^): WC (cm)/height (m)^2^ [24]
- Waist to height cubic (W/Ht^3^, cm/m^3^): WC (cm)/height (m)^3^ [24]
- Height cubic to waist cubic (H^3^/W^3^, cm^3^/m^3^): height (m)^3^/WC (cm)^3^ [24]
- Waist corrected BMI (wBMI, kg/m): [WC (m)][BMI (kg/m^2^)] [25].
- Ponderal index (PI): weight (kg)/height^3^ (cm) [20].
- Body roundness index (BRI): 364.2 − 365.5 × {1 − [(WC (m)/2*π*)/(0.5 × height (m)]^2^}^0.5^ [17–19]
- Body adiposity index (BAI): {hip circumference (cm)/height (m)^1.5^} − 18 [18, 20, 23].
- A body shape index (ABSI, m^11/6^kg^−2/3^): WC (m)/[BMI^2/3^ (kg/m^2^)] [height^1/2^ (m)] [17, 23].
- Conicity index (CI, AU): WC (m)/[0.109√ [weight (kg)/height (m)] [18, 20].
- Body fat distribution index (BFDI, m): [WC/height (m)] + [1/height (m)])/WHR [26].
- Abdominal volume index (AVI, L): {2 × WC (cm)^2^ + 0.7× [waist (cm) − hip (cm)^2^]}/1000 [27].
- Pulse mass index (PMI): (pulse) [BMI (kg/m^2^)]/1.730 [28].

In this study, we defined cutoff values for emergent anthropometric indices to evaluate the association with preHTN and HTN. Values above the second tertile were considered a risk category for BRI (≥4.39), wBMI (≥22.25 kg/m^2^), and PI (≥16.16). The values above the third tertile were considered as a risk category for WHHR (≥0.60 cm), AWI (≥3.25 cm), HWrI (≥6.52 cm), WWrI (≥5.93 cm), W/Ht^2^ (≥40.37 cm/m^2^), W/Ht^3^ (≥26.40 cm/m^3^), BAI (≥36.72), ABSI (≥0.103 m^11/6^ kg^−2/3^), CI (≥1.32 UA), BFDI (≥68.23 m), and AVI (≥18.81 L). Meanwhile, for PMI, a value >1 was considered as a risk category [28].

The detection of high blood pressure was defined according to the Seventh Joint National Committee on prevention, detection, evaluation, and treatment of high blood pressure (JNC7). The following systolic blood pressure (SBP)/diastolic blood pressure (DBP) values were considered and classified: normal blood pressure (SBP/DBP values < 120/80 mmHg), preHTN (SBP/DBP values of 120–139/80–89 mmHg), and HTN (SBP/DBP values ≥ 140/90 mmHg). We also consider those who have antihypertensive treatment as participants with HTN [29].

### 2.4. Statistical Analysis

The statistical analysis of the data was performed using the STATAv.13.0 (StatCorp College Station, TX, USA) and GraphPad Prism v.8.0 software (GraphPad Software, San Diego, CA, USA) for Windows. The categorical variables were compared with the chi-squared test (*X*^2^). Descriptive analysis included the estimation of median and percentiles (5th–95th) for nonparametric variables and determining significant differences among the groups using the Mann–Whitney *U* test. The comparison of median and standard deviation (±) was evaluated using the Student's *t* test. Comparisons between men and women were carried out by independent analysis. The linear relations between anthropometric indices and blood pressure were determined using Spearman correlation coefficient. The association among traditional and new anthropometric indices and preHTN and HTN was calculated using logistic regression analysis in a model adjusted by age, determining the odds ratio (OR) and 95% confidence interval (95% CI). To determine the predictive value of the emergent anthropometric indices for preHTN and HTN, receiving operating characteristics (ROC) were performed. In all cases, the areas under the curve (AUC) and the level of statistical significance or standard error were calculated. A *p* value < 0.05 was considered statistically significant.

## 3. Results

A total of 1,150 participants were evaluated. Traditional factors of CVR such as tobacco (16.11% vs. 3.52%; *p* < 0.001) and alcohol (44.97% vs. 17.84%; *p* < 0.001) consumption were more frequent in men than in women. Women were found to be more sedentary compared to men (43.54% vs. 22.15%; *p* < 0.001, respectively). In general, 48.17% presented abdominal obesity, and according to BMI, 43.74% were overweight and 28.17% presented obesity. A total of 29.74% presented preHTN, and 14.35% presented HTN. The summarized data are presented in Table 1. BMI, AWI, CI, and DBP parameters did not show significant differences between men and women.

In this study, we analyzed the linear relation of SBP and DBP with the emergent anthropometric indices. In the total sample, the AVI (*r* = 0.40, *p* < 0.001), BRI (*r* = 0.37, *p* < 0.001), and wBMI (*r* = 0.36, *p* < 0.001) were strongly correlated with SBP, while for DBP those correlated were BRI (*r* = 0.36, *p* < 0.001) and AVI (*r* = 0.35, *p* < 0.001). The HWrI marker was the only one not related to blood pressure. On the other hand, according to sex, in both men and women, the BRI was more strongly correlated with blood pressure (in men, *r* = 0.36, *p* < 0.001 for SBP, and *r* = 0.37, *p* < 0.001 for DBP; and in women *r* = 0.42, *p* < 0.001 for SBP, and *r* = 0.37, *p* < 0.001 for DBP).

The total sample evaluated in this study was arranged in a logistic regression model adjusted by age and sex. The emergent anthropometric indices associated with preHTN were as follows: BRI (OR = 2.08, 95% CI: 1.49–2.91; *p* < 0.001) and CI (OR = 1.37, 95% CI: 1.00–1.86; *p*=0.044). Meanwhile, for HTN, the main anthropometric indices associated were as follows: CI (OR = 2.47, 95% CI: 1.66–3.69; *p* < 0.001), followed by W/Ht^2^ (OR = 2.19, 95% CI: 1.45–3.31; *p* < 0.001), AVI (OR = 2.02, 95% CI: 1.36–3.01; *p* < 0.001), and PMI (OR = 1.63, 95% CI: 1.04–2.57; *p*=0.032).

**Figure 1:**
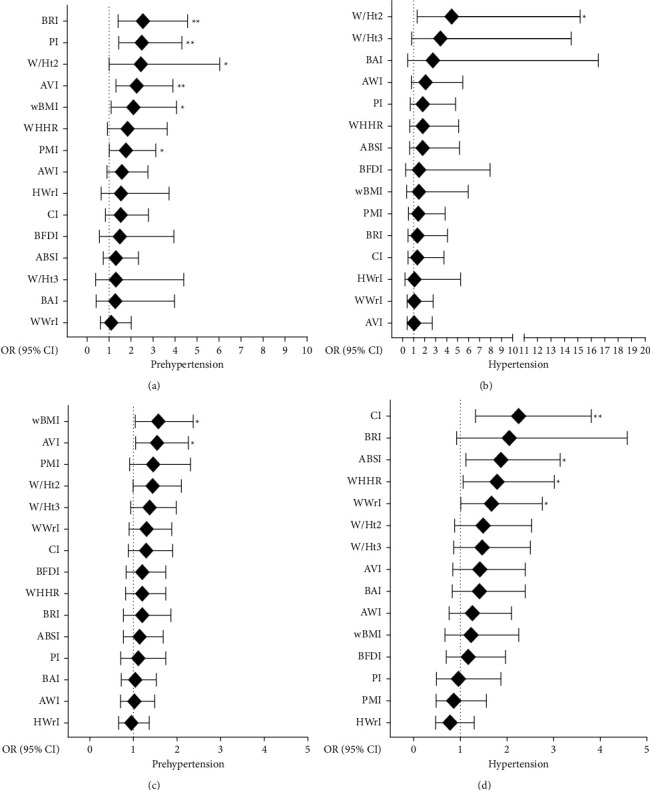
Association of emergent anthropometric indices with preHTN and HTN in men (a, b) and women (c, d). ABSI, a body shape index; AVI, abdominal volume index; AWI, arm-waist index; BAI, body adiposity index; BFDI, body fat distribution index; BRI, body roundness index; CI, conicity index; Ht^3^/W^3^, height cubic to waist cubic; HWrI, hip-wrist index; PI, ponderal index; PMI, pulse mass index; wBMI, waist corrected BMI; WWrI, waist-wrist index; WHHR, waist to hip to height ratio; W/Ht^2^, waist to height square; W/Ht^3^, waist to height cubic. Data shown represent the odds ratio (confidential interval of 95%). Model adjusted by age. ^*∗*^*p* value <0.05 and ^*∗∗*^*p* ≤ 0.01 are statistically significant.

Nevertheless, the analysis according to sex, adjusted by age, proved that in men BRI (OR = 2.54, 95% CI: 1.41–4.57; *p*=0.002), PI (OR = 2.49, 95% CI: 1.44–4.31; *p*=0.001), and W/Ht^2^ (OR = 2.45, 95% CI: 1.00–6.03; *p*=0.050) were mainly associated with preHTN. However, only W/Ht^2^ (OR = 4.46, 95% CI: 1.31–15.17; *p*=0.016) was associated with HTN (Figures 1(a) and 1(b)). On the other hand, for women, wBMI (OR = .57, 95% CI: 1.04–2.37; *p*=0.032) and AVI (OR = 1.54, 95% CI: 1.05–2.26; *p*=0.026) were associated with preHTN, while CI (OR = 2.25, 95% CI: 1.33–3.81; *p*=0.002), ABSI (OR = 1.87, 95% CI: 1.12–3.14; *p*=0.016), WHHR (OR = 1.79, 95% CI: 1.06–3.02; *p*=0.028), and WWrI were associated with HTN (OR = 1.67, 95% CI: 1.01–2.76; *p*=0.045) (Figures 1(c) and 1(d)).

In Table 2, the analysis using ROC curves adjusted by age in the total sample proved that AVI (AUC = 0.645) and CI (AUC = 0.692) showed a moderate predictive value for preHTN and HTN, respectively. In supplementary Table S1, we observed that VF (AUC = 0.680) and WHtR (AUC = 0.656) had a moderate predictive value for preHTN and HTN, respectively.

In Table 3, the indicator for predicting preHTN in men and women was BRI. However, differential indices for HTN were observed between men and women. The principal predictors for HTN in men were ABSI (AUC = 0.777) and W/Ht^3^ (AUC = 0.771), while in women the principal predictors for HTN were CI (AUC = 0.737) and ABSI (AUC = 0.729). These emergent indices have shown a better predictive value than traditional parameters such as VF (AUC = 0.674 and 0.681) and WHtR (AUC = 0.661 and 0.635) among men and women (Supplementary Table S1).

When we evaluated the predictive value of the indices for preHTN and HTN according to sex and age range, we proved that in young men (≤40 years old) the predictor of preHTN was AVI (AUC = 0.680). Meanwhile, for HTN, the predictor was BRI (AUC = 0.702). On the other hand, for women in the same age range, the best predictor for preHTN was wBMI (AUC = 0.672), while for HTN it was PMI (AUC = 0.909) (Table 4). In Supplementary Table S2, we observed that WC (AUC = 0.682) shows a similar predictive value for preHTN in comparison with AVI.

Conversely, in men >40 years old, the best predictors of preHTN were W/Ht^3^ (AUC = 0.670) and W/Ht^2^ (AUC = 0.667), while for HTN, the best predictors were W/Ht^3^ (AUC = 0.748) and W/Ht^2^ (AUC = 0.730). Meanwhile, in women >40 years old, the best predictor of preHTN was CI (AUC = 0.580), while for HTN, the best predictors were ABSI (AUC = 0.685) and CI (AUC = 0.683) (Table 5). W/Ht^3^ appears to represent an emergent index in men >40 years old better than WHtR for preHTN and HTN. While in women >40 years old, the CI and ABSI represent better predictors than VF and WHR (Supplementary Table S2).

## 4. Discussion

The predictive value for preHTN and HTN of sixteen emergent anthropometric indices was demonstrated in this study. The main finding was that the body fat distribution indices had the highest predictive power for preHTN and HTN. However, a differential predictive value of CI, PMI, W/Ht^3^, W/Ht^2^, and ABSI was observed for preHTN and HTN between men and women according to age range.

In this study, the prevalence of preHTN is similar to that reported by Guzmán-Guzmán et al. [2] and lower than the one reported by Rodríguez-Reyes et al. [7]. Campos-Nonato et al. [9] reported 49.2% of HTN in Mexican population. Similar data are reported in several countries. The prevalence of HTN in Indian population, US adults, and Shandong province in China is 40.6% [30], 46.1% [31], and 55.1% [32], respectively. On the other hand, Kibria et al. [33] reported comparable data for the preHTN and HTN in the 2016 Nepal Demographic and Health Survey. The principal factors associated with the development of preHTN and HTN were older age, higher BMI index, high levels of total cholesterol and triglycerides, drinking habits [32, 33], and factors related to central obesity and body fat [2].

In Mexican women, traditional anthropometric indices such as the WC and BMI have been considered the better predictors of CVR, whereas the WHR is a better predictor in men [34]. A cross-sectional study of Italian patients from the Department of Preventive Cardiology proved that the emergent anthropometric index wBMI, along with BMI, WC, and WHtR are related to the patterns of adverse cardiac remodelling, increased arterial stiffness, insulin resistance, and an unfavourable lipid profile [25]. In this sense, the traditional anthropometric indices such as WHtR have been considered the best predictors of at least one cardiometabolic disorder, such as HTN, type 2 diabetes (T2D), metabolic syndrome, dyslipidemia, and hyperuricemia in both sexes [15]. Choi et al. [17] found that the WHtR and BRI displayed an equal predictive power for HTN. In this context, Chang et al. [14] reported that emergent anthropometric indices such as ABSI and BRI are associated with HTN. These data are consistent with our study. Moreover, Adegoke et al. [16] reported that indices of central adiposity (AVI, WC, WHtR, and BRI) were the strongest predictors of HTN. For preHTN, the most strongly related indices are WC, BMI, and WHtR, and PI [20, 35]. However, despite the simplicity and economic ease to evaluate these emergent anthropometric indices, not all of them are of common use in population's screening for CVR factors.

This study showed that emergent anthropometric indices such as BRI, as well as CI, W/Ht^2^, and AVI are associated with the presence of preHTN and HTN. However, CI was consistently correlated with SBP and DBP despite the moderate predictive value in preHTN, while PMI was an emergent index related to HTN, principally in women ≤40 years old. A previous study demonstrated that CI is mostly associated with the development of HTN in men [18], similar to the data shown in our study, whereas in women, CI has been correlated with SBP (*r* = 0.29; *p* < 0.01) and DBP (*r* = 0.20; *p* < 0.01). It has been identified in urban women from Delhi [19] and Nigeria (SBP, *r* = 0.114; *p* < 0.01 and DBP, *r* = 0.119; *p* < 0.01) [16] as well as with SBP (*r* = 0.22; *p* < 0.05) in menopausal women from Iran [36], and with HTN and T2D in women from Brazil [18, 37]. Although it has been proven that CI has a higher predictive value for HTN in women [38] than in adult men [39], it appears to have a limiting predictive value in young populations [40]. These results are in concordance with our findings in women ≥40 years old, in which potentially hormonal changes related to decreasing estrogen levels also contribute to fat gain and redistribution of total body fat toward the central abdominal region, revealing the loss of the cardioprotection status that characterizes the incidence of HTN related to sexual dimorphism [41, 42]. Also, a cross-sectional study in adults from northern Iran determined that CI had a more discriminatory accuracy for 10-year-old cardiovascular events compared to WC, WHtR, and AVI [43], and for coronary risk [44]. Therefore, future research should evaluate the effect of CI on blood pressure in a variable context of sex and age, ancestry, and nutritional factors in different populations.

In the present study, according to the prediction value, ABSI > W/Ht^3^>W/Ht^2^>CI showed predictive ability for HTN, while BRI > W/Ht^2^>AVI > PI for preHTN in men. In other studies, the emergent indices reported as predictors for HTN in men were BAI > WHHR > ABSI [23], BRI > ABSI [13, 14], AVI > BRI [16], CI > BAI [18], CI [45, 46], and WrC [47], whereas for preHTN it was PI > BAI [20]. The predictive value for ABSI and CI was consistent between populations [14, 17, 38, 39]. However, our study shows that the W/Ht^3^ and W/Ht^2^ indices have a potential predictive value for HTN, while that W/Ht^2^ and PI were consistent markers for preHTN and could be useful for screening impaired blood pressure in the population with similar somatometric characteristics.

Interestingly, in this study, PMI was associated with preHTN and HTN. PMI is considered an emergent CVR marker. Koch et al. [48] reported that a PMI >1 is related to susceptibility to suffering CVD over a period of 5 years. In women with generalized lupus erythematosus, the PMI was considered a predictor of CVD [49]. The relation of PMI on the CVD prognostic can be associated with preHTN and development of HTN mainly in young women. Besides, in women, we found that CI > ABSI > W/Ht^2^ have predictive power for HTN, while that BRI > AVI > W/Ht^2^>CI for preHTN. Other emergent anthropometric indices related to adiposity have been consistently reported as predictors of HTN in women of other populations, among them we found that BAI > WHHR > ABSI [23], BRI > ABSI [13, 14], AVI > BRI [16], CI > BAI [18], CI [39, 45, 46, 50], and PI > BAI for preHTN [20]. The predictive value of indices related to central obesity can be explained by several mechanisms, including the biological function of adipokines and cytokines, such as adiponectin, tumor necrosis factor-*α*, and leptin, principally in woman [51] by the stimulation of the renin-angiotensin system, considered one of the essential mechanisms in obesity-related HTN [52].

In this study, we observed that age and being male are biological variables associated with HTN and preHTN and have a differential predictive value for anthropometric indices. It is important to consider that these indicators are easy to measure, noninvasive, and inexpensive, and their clinical value is relevant, so they should be included as routine evaluations in clinics and health campaigns. It is also important to emphasize that the specific use of indices according to sex and age range could increase the screening for risk of preHTN and HTN in populations and could be useful for implementing intervention measures. The limitation of this study was that the measurements of biochemical-metabolic parameters were not included. Furthermore, the cutoff values for risk categories in this study can vary in other populations.

## 5. Conclusions

In conclusions, the body adiposity distribution indices predict preHTN in men and women. Nevertheless, emergent indices such as CI, PMI, W/Ht^3^, W/Ht^2^, and ABSI could represent differential predictors of preHTN and HTN according to sex and age range and could be implemented to perform high blood pressure screenings in the population.

## Figures and Tables

**Table 1:** Anthropometrics and clinical characteristics of the study population according to sex.

Variables	Total (*n* = 1150)	Men (*n* = 298)	Women (*n* = 852)	*p* value
Age (years)^a^	42 (20–66)	41 (18–70)	43 (21–65)	0.022
Height (m)^a^	1.55 (1.43–1.74)	1.67 (1.56–1.80)	1.52 (1.42–1.64)	<0.001
Weight (kg)^a^	66.1 (49.3–92.6)	75.55 (56.7–98.9)	63.45 (48.4–85.5)	<0.001
WC (cm)^a^	92 (74–112)	96 (75–115)	90 (73–112)	<0.001
BMI (kg/m^2^)^a^	27.4 (20.9–36.7)	27.5 (20.7–34.9)	27.3 (21–37.1)	0.80
WHR (cm)^a^	0.90 (0.80–1.01)	0.94 (0.82–1.05)	0.89 (0.79–0.99)	<0.001
WHtR (cm)^a^	0.58 (0.45–0.73)	0.57 (0.44–0.69)	0.59 (0.47–0.74)	<0.001
Body fat (%)^a^	39.9 (20.7–52.1)	28.2 (15.1–39.1)	42.5 (29.8–52.8)	<0.001
Visceral fat (%)^a^	8 (4–16)	11 (3–20)	8 (4–13)	<0.001

*Emergent anthropometric indices*				
AWI (cm)^a^	3.12 (2.70–3.68)	3.13 (2.71–3.69)	3.12 (2.70–3.68)	0.47
HWrI (cm)^a^	6.29 (5.42–7.34)	5.91 (5.35–6.68)	6.42 (5.56–7.42)	<0.001
WWrI (cm)^a^	5.69 (4.73–6.68)	5.61 (4.58–6.40)	5.71 (4.77–6.76)	<0.001
WHHR (m^−1^)^a^	0.57 (0.49–0.66)	0.56 (0.47–0.65)	0.58 (0.50–0.67)	<0.001
W/Ht^2^ (cm/m^2^)^a^	37.46 (28.39–49.79)	33.88 (25.71–43.01)	38.83 (29.93–50.63)	<0.001
W/Ht^3^ (cm/m^3^)^a^	23.96 (16.99–34.19)	20.50 (14.86–27.33)	25.48 (18.58–35.16)	<0.001
Ht^3^/W^3^ (cm^3^/m^3^)^b^	5.43 ± 2.37	5.94 ± 2.54	5.25 ± 2.29	<0.001
wBMI (kg/m)^a^	25.11 (15.90–40.65)	26.74 (16.03–40.35)	24.65 (15.75–40.65)	0.009
PI^a^	17.53 (12.99–24.28)	16.34 (11.95–21.07)	17.90 (13.31–24.82)	<0.001
BRI^a^	5.10 (2.63–8.88)	4.81 (2.35–7.77)	5.23 (2.83–9.23)	<0.001
BAI^a^	33.54 (24.76–47.31)	28.59 (22.05–37.26)	35.44 (26.90–48.09)	<0.001
ABSI (m^11/6^ kg^−2/3^)^a^	0.100 (0.090–0.111)	0.101 (0.091–0.110)	0.100 (0.090–0.111)	0.031
CI (AU)^a^	1.28 (1.14–1.42)	1.29 (1.14–1.42)	1.28 (1.14–1.43)	0.06
BFDI (m)^a^	64.96 (55.76–80.08)	61.20 (53.30–70.91)	66.91 (57.31–81.40)	<0.001
AVI (L)^a^	16.92 (10.95–25.08)	18.43 (11.40–26.45)	16.20 (10.65–25.08)	<0.001
PMI^a^	1.14 (0.79–1.65)	1.11 (0.78–1.61)	1.15 (0.80–1.65)	0.027

*Blood pressure measures*				
SBP (mmHg)^a^	113 (91–152)	119 (95–151)	112 (90–152)	<0.001
DBP (mmHg)^a^	74 (57–94)	75 (55–93)	74 (57–94)	0.83
Blood pressure category^c^				<0.001
Normotensive	643 (55.91)	146 (48.99)	497 (58.33)	
Prehypertension	342 (29.74)	111 (37.25)	231 (27.11)	
Hypertension	165 (14.35)	41 (13.76)	124 (14.55)	

ABSI, a body shape index; AVI, abdominal volume index; AWI, arm-waist index; BAI, body adiposity index; BFDI, body fat distribution index; BMI, body mass index; BRI, body roundness index; CI, conicity index; DBP, diastolic blood pressure; Ht^3^/W^3^, height cubic to waist cubic; HWrI, hip-wrist index; PI, ponderal index; PMI, pulse mass index; SBP, systolic blood pressure; wBMI, waist corrected BMI; WC, waist circumference; WWrI, waist-wrist index; WHHR, waist to hip to height ratio; WHR, waist to hip ratio; W/Ht^2^, waist to height square; W/Ht^3^, waist to height cubic; WHtR, waist to height ratio. Data shown represent ^a^median and percentile (p5th-p95th), ^b^mean and standard deviation and ^c^proportions (%). *p* value <0.05 is statistically significant.

**Table 2:** Predictive value of the emergent anthropometric indices to preHTN and HTN in the total sample.

Variables	PreHTN (AUC, 95% CI)	Variables	HTN (AUC, 95% CI)
AVI (L)	0.645 (0.640–0.651)^*∗*^^*∗*^	CI (UA)	0.692 (0.686–0.698)^*∗*^^*∗*^
wBMI (kg/m)	0.641 (0.635–0.646)^*∗*^^*∗*^	ABSI (m^11/6^ kg^−2/3^)	0.687 (0.681–0.693)^*∗*^^*∗*^
BRI	0.634 (0.628–0.639)^*∗*^^*∗*^	BRI	0.656 (0.650–0.662)^*∗*^^*∗*^
CI (UA)	0.616 (0.611–0.622)^*∗*^^*∗*^	WHHR (m^−1^)	0.653 (0.647–0.660)^*∗*^^*∗*^
PI	0.611 (0.605–0.616)^*∗*^^*∗*^	W/Ht^2^ (cm/m^2^)	0.648 (0.641–0.654)^*∗*^^*∗*^
W/Ht^2^ (cm/m^2^)	0.607 (0.602–0.613)^*∗*^^*∗*^	W/Ht^3^ (cm/m^3^)	0.634 (0.628–0.641)^*∗*^^*∗*^
WHHR (m^−1^)	0.600 (0.595–0.606)^*∗*^^*∗*^	AVI (L)	0.630 (0.624–0.636)^*∗*^^*∗*^
ABSI (m^11/6^ kg^−2/3^)	0.589 (0.583–0.595)^*∗*^^*∗*^	AWI (cm)	0.615 (0.608–0.621)^*∗*^^*∗*^
W/Ht^3^ (cm/m^3^)	0.585 (0.579–0.591)^*∗*^^*∗*^	WWrI (cm)	0.602 (0.596–0.608)^*∗*^^*∗*^
WWrI (cm)	0.582 (0.577–0.588)^*∗*^^*∗*^	BAI	0.597 (0.591–0.603)^*∗*^^*∗*^
BFDI (m)	0.579 (0.573–0.585)^*∗*^^*∗*^	BFDI (m)	0.594 (0.588–0.601)^*∗*^^*∗*^
BAI	0.568 (0.563–0.574)^*∗*^^*∗*^	wBMI (kg/m)	0.588 (0.582–0.594)^*∗*^^*∗*^
PMI	0.559 (0.554–0.565)^*∗*^^*∗*^	PI	0.584 (0.577–0.590)^*∗*^^*∗*^
AWI (cm)	0.551 (0.545–0.557)^*∗*^^*∗*^	PMI	0.513 (0.506–0.519)^*∗*^^*∗*^
HWrI (cm)	0.496 (0.490–0.502)^*∗*^^*∗*^	HWrI (cm)	0.499 (0.493–0.506)^*∗*^^*∗*^

ABSI, a body shape index; AVI, abdominal volume index; AWI, arm-waist index; BAI, body adiposity index; BFDI, body fat distribution index; BRI, body roundness index; CI, conicity index; Ht^3^/W^3^, height cubic to waist cubic; HWrI, hip-wrist index; PI, ponderal index; PMI, pulse mass index; wBMI, waist corrected BMI; WWrI, waist-wrist index; WHHR, waist to hip to height ratio; W/Ht^2^, waist to height square; W/Ht^3^, waist to height cubic. Data shown are the receiver operating characteristic distribution of the areas under curves considering the criterion variables prehypertension (SBP: 120–139 mmHg/DBP: 80–89 mmHg) and hypertension (SBP: ≥140 mmHg/DBP: ≥90 mmHg). Adjusted by age. ^*∗∗*^*p* value ≤0.01.

**Table 3:** Predictive value of the emergent anthropometric indices of preHTN and HTN according to sex.

Variables	PreHTN (AUC, 95% CI)	Variables	HTN (AUC, 95% CI)
*Men*			
BRI	0.661 (0.650–0.672)^*∗*^^*∗*^	ABSI (m^11/6^kg^−2/3^)	0.777 (0.765–0.788)^*∗*^^*∗*^
W/Ht^2^ (cm/m^2^)	0.653 (0.642–0.664)^*∗*^^*∗*^	W/Ht^3^ (cm/m^3^)	0.771 (0.759–0.783)^*∗*^^*∗*^
AVI (L)	0.650 (0.639–0.661)^*∗*^^*∗*^	W/Ht^2^ (cm/m^2^)	0.767 (0.754–0.779)^*∗*^^*∗*^
PI	0.648 (0.637–0.658)^*∗*^^*∗*^	CI (UA)	0.766 (0.755–0.788)^*∗*^^*∗*^
wBMI (kg/m)	0.645 (0.634–0.655)^*∗*^^*∗*^	WHHR (m^−1^)	0.752 (0.739–0.766)^*∗*^^*∗*^
WHHR (m^−1^)	0.639 (0.628–0.650)^*∗*^^*∗*^	BRI	0.742 (0.729–0.754)^*∗*^^*∗*^
CI (UA)	0.639 (0.628–0.649)^*∗*^^*∗*^	BAI	0.727 (0.714–0.739)^*∗*^^*∗*^
W/Ht^3^ (cm/m^3^)	0.637 (0.626–0.648)^*∗*^^*∗*^	AWI (cm)	0.722 (0.709–0.735)^*∗*^^*∗*^
WWrI (cm)	0.628 (0.617–0.639)^*∗*^^*∗*^	BFDI (m)	0.709 (0.697–0.721)^*∗*^^*∗*^
ABSI (m^11/6^kg^−2/3^)	0.620 (0.609–0.631)^*∗*^^*∗*^	AVI (L)	0.673 (0.660–0.685)^*∗*^^*∗*^
BAI	0.618 (0.607–0.629)^*∗*^^*∗*^	PI	0.685 (0.672–0.698)^*∗*^^*∗*^
BFDI (m)	0.617 (0.606–0.628)^*∗*^^*∗*^	wBMI (kg/m)	0.639 (0.626–0.652)^*∗*^^*∗*^
AWI (cm)	0.608 (0.597–0.619)^*∗*^^*∗*^	WWrI (cm)	0.634 (0.619–0.648)^*∗*^^*∗*^
HWrI (cm)	0.544 (0.533–0.555)^*∗*^^*∗*^	PMI	0.487 (0.474–0.501)^*∗*^^*∗*^
PMI	0.506 (0.495–0.517)^*∗*^^*∗*^	HWrI (cm)	0.510 (0.495–0.525)^*∗*^^*∗*^

*Women*			
BRI	0.635 (0.628–0.641)^*∗*^^*∗*^	CI (UA)	0.737 (0.730–0.744)^*∗*^^*∗*^
AVI (L)	0.630 (0.623–0.636)^*∗*^^*∗*^	ABSI (m^11/6^kg^−2/3^)	0.729 (0.721–0.736)^*∗*^^*∗*^
W/Ht^2^ (cm/m^2^)	0.628 (0.621–0.634)^*∗*^^*∗*^	W/Ht^2^ (cm/m^2^)	0.712 (0.705–0.720)^*∗*^^*∗*^
CI (UA)	0.624 (0.617–0.631)^*∗*^^*∗*^	W/Ht^3^ (cm/m^3^)	0.709 (0.702–0.717)^*∗*^^*∗*^
wBMI (kg/m)	0.618 (0.612–0.625)^*∗*^^*∗*^	BRI	0.708 (0.701–0.715)^*∗*^^*∗*^
W/Ht^3^ (cm/m^3^)	0.618 (0.611–0.625)^*∗*^^*∗*^	WHHR (m^−1^)	0.702 (0.694–0.710)^*∗*^^*∗*^
BFDI (m)	0.611 (0.604–0.617)^*∗*^^*∗*^	AVI (L)	0.678 (0.670–0.685)^*∗*^^*∗*^
BAI	0.608 (0.601–0.614)^*∗*^^*∗*^	BAI	0.669 (0.661–0.677)^*∗*^^*∗*^
WHHR (m^−1^)	0.603 (0.596–0.610)^*∗*^^*∗*^	BFDI (m)	0.653 (0.645–0.661)^*∗*^^*∗*^
ABSI (m^11/6^kg^−2/3^)	0.600 (0.593–0.607)^*∗*^^*∗*^	WWrI (cm)	0.640 (0.632–0.648)^*∗*^^*∗*^
PI	0.599 (0.593–0.606)^*∗*^^*∗*^	AWI (cm)	0.631 (0.622–0.639)^*∗*^^*∗*^
WWrI (cm)	0.574 (0.567–0.581)^*∗*^^*∗*^	PI	0.624 (0.616–0.632)^*∗*^^*∗*^
PMI	0.545 (0.538–0.552)^*∗*^^*∗*^	wBMI (kg/m)	0.623 (0.616–0.631)^*∗*^^*∗*^
AWI (cm)	0.537 (0.530–0.544)^*∗*^^*∗*^	PMI	0.505 (0.496–0.514)^*∗*^^*∗*^
HWrI (cm)	0.509 (0.502–0.516)^*∗*^^*∗*^	HWrI (cm)	0.497 (0.488–0.505)^*∗*^^*∗*^

ABSI, a body shape index; AVI, abdominal volume index; AWI, arm-waist index; BAI, body adiposity index; BFDI, body fat distribution index; BRI, body roundness index; CI, conicity index; Ht^3^/W^3^, height cubic to waist cubic; HWrI, hip-wrist index; PI, ponderal index; PMI, pulse mass index; wBMI, waist corrected BMI; WWrI, waist-wrist index; WHHR, waist to hip to height ratio; W/Ht^2^, waist to height square; W/Ht^3^, waist to height cubic. Data shown are the receiver operating characteristic distribution of the areas under curves considering the criterion variables prehypertension (SBP: 120–139 mmHg/DBP: 80–89 mmHg) and hypertension (SBP: ≥140 mmHg/DBP: ≥90 mmHg). Adjusted by age. ^*∗∗*^*p* value ≤0.01.

**Table 4:** Predictive value of the emergent anthropometric indices for preHTN and HTN by gender in ≤40 years old.

Variables	PreHTN (AUC, 95% CI)	Variables	HTN (AUC, 95% CI)
*Men ≤40 years old*			
AVI (L)	0.680 (0.581–0.779)^*∗*^	BRI	0.702 (0.605–0.799)^*∗*^
WWrI (cm)	0.655 (0.555–0.755)^*∗*^	W/Ht^2^ (cm/m^2^)	0.689 (0.595–0.783)^*∗*^
HWrI (cm)	0.654 (0.553–0.756)^*∗*^	wBMI (kg/m)	0.702 (0.565–0.839)
CI (UA)	0.650 (0.549–0.750)^*∗*^	PI	0.683 (0.430–0.935)
wBMI (kg/m)	0.650 (0.546–0.754)^**†**^	AVI (L)	0.679 (0.578–0.780)^*∗*^
BRI	0.624 (0.518–0.731)^**†**^	WHHR (m^−1^)	0.666 (0.497–0.835)
ABSI (m^11/6^kg^−2/3^)	0.621 (0.514–0.728)^**†**^	PMI	0.640 (0.125–1.000)
BFDI (m)	0.604 (0.499–0.709)^*∗*^	W/Ht^3^ (cm/m^3^)	0.634 (0.536–0.731)^*∗*^
PI	0.588 (0.476–0.701)	BFDI (m)	0.617 (0.473–0.761)
AWI (cm)	0.587 (0.478–0.695)^**†**^	BAI	0.611 (0.452–0.769)
W/Ht^2^ (cm/m^2^)	0.568 (0.459–0.677)^**†**^	CI (UA)	0.588 (0.172–1.000)
BAI	0.560 (0.453–0.667)^**†**^	AWI (cm)	0.585 (0.207–0.962)
PMI	0.557 (0.454–0.661)^*∗*^	ABSI (m^11/6^kg^−2/3^)	0.565 (0.061–1.000)
W/Ht^3^ (cm/m^3^)	0.540 (0.396–0.612)^**†**^	WWrI (cm)	0.558 (0.047–1.000)
WHHR (m^−1^)	0.467 (0.361–0.573)^†^	HWrI (cm)	0.521 (0–1.000)

*Women ≤40 years old*			
wBMI (kg/m)	0.672 (0.580–0.763)^*∗*^	PMI	0.909 (0.797–1.000)^**†**^
AVI (L)	0.667 (0.577–0.757)^*∗*^	BFDI (m)	0.772 (0.625–0.918)
PI	0.665 (0.572–0.757)^*∗*^	BAI	0.761 (0.649–0.873)^**†**^
BRI	0.662 (0.569–0.755)^*∗*^	PI	0.743 (0.555–0.931)
PMI	0.650 (0.653–0.736)^*∗*^	BRI	0.734 (0.530–0.939)
BFDI (m)	0.645 (0.555–0.736)^*∗*^	wBMI (kg/m)	0.722 (0.505–0.939)
W/Ht^2^ (cm/m^2^)	0.641 (0.545–0.737)^*∗*^	AVI (L)	0.718 (0.476–0.961)
BAI	0.635 (0.543–0.727)^*∗*^	W/Ht^2^ (cm/m^2^)	0.713 (0.546–0.880)
WWrI (cm)	0.631 (0.542–0.719)^*∗*^	W/Ht^3^ (cm/m^3^)	0.674 (0.511–0.837)
W/Ht^3^ (cm/m^3^)	0.624 (0.523–0.724)^*∗*^	CI (UA)	0.645 (0.378–0.911)
WHHR (m^−1^)	0.604 (0.499–0.709)^**†**^	ABSI (m^11/6^kg^−2/3^)	0.576 (0.299–0.854)
CI (UA)	0.602 (0.512–0.693)^*∗*^	WHHR (m^−1^)	0.552 (0.283–0.820)
HWrI (cm)	0.570 (0.477–0.663)^*∗*^	WWrI (cm)	0.549 (0.275–0.824)
ABSI (m^11/6^kg^−2/3^)	0.568 (0.477–0.659)^*∗*^	HWrI (cm)	0.496 (0.262–0.730)
AWI (cm)	0.561 (0.459–0.664)^*∗*^	AWI (cm)	0.409 (0.170–0.649)

ABSI, a body shape index; AVI, abdominal volume index; AWI, arm-waist index; BAI, body adiposity index; BFDI, body fat distribution index; BRI, body roundness index; CI, conicity index; Ht^3^/W^3^, height cubic to waist cubic; HWrI, hip-wrist index; PI, ponderal index; PMI, pulse mass index; wBMI, waist corrected BMI; WWrI, waist-wrist index; WHHR, waist to hip to height ratio; W/Ht^2^, waist to height square; W/Ht^3^, waist to height cubic. Data shown are the receiver operating characteristic distribution of the areas under curves considering the criterion variables prehypertension (SBP: 120–139 mmHg/DBP: 80–89 mmHg) and hypertension (SBP: ≥140 mmHg/DBP: ≥90 mmHg). Adjusted by age. ^*∗*^*p* value ≤0.05, and ^†^*p*value = 0.05.

**Table 5:** Predictive value of the emergent anthropometric indices for preHTN and HTN by gender in >40 years old.

Variables	PreHTN (AUC, 95% CI)	Variables	HTN (AUC, 95% CI)
*Men >40 years old*			
W/Ht^3^ (cm/m^3^)	0.670 (0.574–0.766)^*∗*^	W/Ht^3^ (cm/m^3^)	0.748 (0.634–0.862)^**†**^
W/Ht^2^ (cm/m^2^)	0.667 (0.570–0.763)^*∗*^	W/Ht^2^ (cm/m^2^)	0.730 (0.610–0.850)
WHHR (m^−1^)	0.659 (0.563–0.755)^*∗*^	ABSI (m^11/6^kg^−2/3^)	0.708 (0.600–0.817)^**†**^
PI	0.636 (0.539–0.734)^*∗*^	BAI	0.708 (0.592–0.823)
BRI	0.636 (0.538–0.733)^*∗*^	WHHR (m^−1^)	0.704 (0.580–0.828)
BAI	0.634 (0.535–0.733)^*∗*^	CI (UA)	0.694 (0.579–0.808)
BFDI (m)	0.606 (0.506–0.706)^*∗*^	BRI	0.684 (0.560–0.807)
wBMI (kg/m)	0.588 (0.487–0.688)^*∗*^	BFDI (m)	0.677 (0.560–0.794)
CI (UA)	0.586 (0.485–0.687)^*∗*^	AWI (cm)	0.656 (0.536–0.775)
AVI (L)	0.585 (0.484–0.686)^*∗*^	PI	0.639 (0.515–0.764)
WWrI (cm)	0.579 (0.477–0.681)^*∗*^	AVI (L)	0.578 (0.455–0.700)
ABSI (m^11/6^kg^−2/3^)	0.564 (0.463–0.666)^*∗*^	wBMI (kg/m)	0.552 (0.426–0.678)
AWI (cm)	0.551 (0.449–0.653)^*∗*^	WWrI (cm)	0.548 (0.419–0.677)
HWrI (cm)	0.515 (0.413–0.618)^*∗*^	HWrI (cm)	0.501 (0.374–0.627)
PMI	0.469 (0.367–0.572)^*∗*^	PMI	0.458 (0.338–0.578)

*Women >40 years old*			
CI (UA)	0.580 (0.521–0.638)^*∗*^	ABSI (m^11/6^kg^−2/3^)	0.685 (0.620–0.749)^*∗*^
AVI (L)	0.569 (0.511–0.627)^*∗*^	CI (UA)	0.683 (0.620–0.746)^*∗*^
BRI	0.569 (0.511–0.627)^*∗*^	W/Ht^3^ (cm/m^3^)	0.641 (0.574–0.709)^*∗*^
ABSI (m^11/6^kg^−2/3^)	0.564 (0.504–0.623)^*∗*^	W/Ht^2^ (cm/m^2^)	0.641 (0.574–0.708)^*∗*^
W/Ht^2^ (cm/m^2^)	0.561 (0.503–0.620)^*∗*^	WHHR (m^−1^)	0.656 (0.589–0.723)^*∗*^
wBMI (kg/m)	0.555 (0.496–0.613)^*∗*^	BRI	0.636 (0.570–0.702)^*∗*^
WHHR (m^−1^)	0.554 (0.496–0.612)^*∗*^	AWI (cm)	0.608 (0.538–0.677)^*∗*^
W/Ht^3^ (cm/m^3^)	0.552 (0.493–0.611)^*∗*^	AVI (L)	0.607 (0.542–0.673)^*∗*^
BFDI (m)	0.540 (0.481–0.598)^*∗*^	WWrI (cm)	0.599 (0.533–0.666)^*∗*^
BAI	0.536 (0.476–0.595)^*∗*^	BAI	0.586 (0.515–0.657)^*∗*^
WWrI (cm)	0.531 (0.471–0.591)^*∗*^	BFDI (m)	0.570 (0.500–0.640)^*∗*^
PI	0.527 (0.468–0.587)^*∗*^	wBMI (kg/m)	0.552 (0.484–0.621)^*∗*^
AWI (cm)	0.509 (0.449–0.569)^*∗*^	PI	0.548 (0.477–0.618)^*∗*^
PMI	0.504 (0.445–0.563)^*∗*^	HWrI (cm)	0.473 (0.402–0.543)^*∗*^
HWrI (cm)	0.480 (0.420–0.539)^*∗*^	PMI	0.468 (0.396–0.540)^*∗*^

ABSI, a body shape index; AVI, abdominal volume index; AWI, arm-waist index; BAI, body adiposity index; BFDI, body fat distribution index; BRI, body roundness index; CI, conicity index; Ht^3^/W^3^, height cubic to waist cubic; HWrI, hip-wrist index; PI, ponderal index; PMI, pulse mass index; wBMI, waist corrected BMI; WWrI, waist-wrist index; WHHR, waist to hip to height ratio; W/Ht^2^, waist to height square; W/Ht^3^, waist to height cubic. Data shown are the receiver operating characteristic distribution of the areas under curves considering the criterion variables prehypertension (SBP: 120–139 mmHg/DBP: 80–89 mmHg) and hypertension (SBP: ≥140 mmHg/DBP: ≥90 mmHg). Adjusted by age. ^*∗*^*p*^*∗*^*p-*value ≤0.05, ^†^*p* value = 0.05.

## Data Availability

The data used to support the findings of the study can be obtained from the corresponding author.

## Supplementary Materials

Supplementary Materials Supplementary Table 1: Predictive value of the traditional anthropometric indices for preHTN and HTN by the total sample and sex. Supplementary Table 2: Predictive value of the traditional anthropometric indices for preHTN and HTN by gender and age.
