# Supplementary material for: Emergent Anthropometric Indices in Differential Prediction of Prehypertension and Hypertension in Mexican Population: Results according to Age and Sex
Source: Int J Hypertens. 2022 Jul 7;2022:4522493. doi: 10.1155/2022/4522493 (PMC9283069; doi:10.1155/2022/4522493)
Supplement: Supplementary Materials — Supplementary Table 1: Predictive value of the traditional anthropometric indices for preHTN and HTN by the total sample and sex. Supplementary Table 2: Predictive value of the traditional anthropometric indices for preHTN and HTN by gender and age. [file 4522493.f1.docx]

## Supplementary Materials

| **Supplementary TABLE 1:** Predictive value of the traditional anthropometric indices for preHTN and HTN by in the total sample and sex | | | | | |
| --- | --- | --- | --- | --- | --- |
| **Variables** | **preHTN (AUC, 95% CI)** | ***p*-value** | **Variables** | **HTN (AUC, 95% CI)** | ***p*-value** |
| Global |  |  |  |  |  |
| VF (%) | 0.680 (0.674-0.685) | 0.002 | WHtR (cm) | 0.656 (0.650-0.662) | 0.002 |
| WC (cm) | 0.645 (0.639-0.650) | 0.002 | VF (%) | 0.652 (0.647-0.658) | 0.003 |
| WHtR (cm) | 0.634 (0.628-0.639) | 0.002 | WC (cm) | 0.629 (0.623-0.635) | 0.003 |
| BMI (kg/m^2^) | 0.627 (0.621-0.632) | 0.002 | WHR (cm) | 0.628 (0.621-0.634) | 0.003 |
| WHR (cm) | 0.618 (0.612-0.623) | 0.002 | BMI (kg/m^2^) | 0.554 (0.547-0.560) | 0.003 |
| BF (%) | 0.525 (0.519-0.531) | 0.003 | BF (%) | 0.543 (0.537-0.550) | 0.003 |
| Men |  |  |  |  |  |
| VF (%) | 0.674 (0.663-0.684) | 0.005 | WHtR (cm) | 0.742 (0.729-0.754) | 0.006 |
| WHtR (cm) | 0.661 (0.650-0.672) | 0.005 | VF (%) | 0.704 (0.691-0.716) | 0.006 |
| WHR (cm) | 0.651 (0.640-0.661) | 0.005 | WHR (cm) | 0.706 (0.692-0.720) | 0.007 |
| WC (cm) | 0.648 (0.637-0.659) | 0.005 | WC (cm) | 0.670 (0.658-0.683) | 0.006 |
| BMI (kg/m^2^) | 0.634 (0.623-0.645) | 0.005 | BMI (kg/m^2^) | 0.613 (0.600-0.627) | 0.006 |
| BF (%) | 0.599 (0.588-0.610) | 0.005 | BF (%) | 0.560 (0.545-0.574) | 0.007 |
| Women |  |  |  |  |  |
| VF (%) | 0.681 (0.675-0.688) | 0.003 | VF (%) | 0.747 (0.740-0.753) | 0.003 |
| WHtR (cm) | 0.635 (0.628-0.641) | 0.003 | WHtR (cm) | 0.708 (0.701-0.715) | 0.003 |
| WC (cm) | 0.630 (0.623-0.636) | 0.003 | WHR (cm) | 0.679 (0.671-0.687) | 0.004 |
| BMI (kg/m^2^) | 0.599 (0.592-0.606) | 0.003 | WC (cm) | 0.676 (0.669-0.638) | 0.003 |
| WHR (cm) | 0.598 (0.591-0.604) | 0.003 | BMI (kg/m^2^) | 0.580 (0.572-0.589) | 0.004 |
| BF (%) | 0.567 (0.560-0.574) | 0.003 | BF (%) | 0.573 (0.565-0.581) | 0.004 |
| BF, body fat; BMI, body mass index; VF, visceral fat; WC, waist circumference; WHR, waist to hip ratio; WHtR, waist to height ratio.  Data shown Receiver Operating Characteristics Distribution of the areas under curves considering the criterion variable the prehypertension (SBP: 120-139 mmHg/DBP: 80-89 mmHg) and hypertension (SBP: ≥140 mmHg/DBP: ≥90 mmHg). Adjusted by age. | | | | | |

| **Supplementary TABLE 2:** Predictive value of the traditional anthropometric indices for preHTN and HTN by gender and age | | | | | |
| --- | --- | --- | --- | --- | --- |
| **Variables** | **PreHTN (AUC, 95% CI)** | ***p*-value** | **Variables** | **HTN (AUC, 95% CI)** | ***p*-value** |
| Men ≤40 years old |  |  |  |  |  |
| WC (cm) | 0.682 (0.584-0.780) | 0.050 | VF (%) | 0.782 (0.624-0.941) | 0.080 |
| WHtR (cm) | 0.624 (0.518-0.731) | 0.054 | BF (%) | 0.719 (0.554-0.883) | 0.084 |
| BMI (kg/m^2^) | 0.620 (0.511-0.729) | 0.055 | WHR (cm) | 0.715 (0.533-0.898) | 0.093 |
| BF (%) | 0.618 (0.516-0.720) | 0.052 | WHtR (cm) | 0.702 (0.605-0.799) | 0.049 |
| VF (%) | 0.618 (0.507-0.729) | 0.056 | BMI (kg/m^2^) | 0.687 (0.464-0.911) | 0.114 |
| WHR (cm) | 0.575 (0.468-0.681) | 0.054 | WC (cm) | 0.679 (0.576-0.783) | 0.052 |
| Men >40 years old |  |  |  |  |  |
| WHtR (cm) | 0.636 (0.538-0.733) | 0.049 | WHtR (cm) | 0.684 (0.560-0.807) | 0.063 |
| VF (%) | 0.634 (0.536-0.732) | 0.050 | WHR (cm) | 0.632 (0.501-0.764) | 0.067 |
| WHR (cm) | 0.626 (0.527-0.725) | 0.050 | VF (%) | 0.587 (0.462-0.712) | 0.063 |
| BMI (kg/m^2^) | 0.591 (0.491-0.691) | 0.051 | WC (cm) | 0.576 (0.453-0.698) | 0.062 |
| WC (cm) | 0.583 (0.482-0.684) | 0.051 | BMI (kg/m^2^) | 0.541 (0.415-0.668) | 0.064 |
| BF (%) | 0.565 (0.464-0.667) | 0.051 | BF (%) | 0.516 (0.381-0.650) | 0.068 |
| Women ≤40 years old |  |  |  |  |  |
| VF (%) | 0.679 (0.586-0.772) | 0.047 | WHtR (cm) | 0.734 (0.530-0.939) | 0.104 |
| BMI (kg/m^2^) | 0.670 (0.579-0.762) | 0.046 | BF (%) | 0.729 (0.555-0.903) | 0.088 |
| WC (cm) | 0.667 (0.577-0.757) | 0.045 | BMI (kg/m^2^) | 0.726 (0.512-0.940) | 0.109 |
| WHtR (cm) | 0.662 (0.569-0.755) | 0.047 | WC (cm) | 0.722 (0.485-0.959) | 0.121 |
| BF (%) | 0.633 (0.533-0.732) | 0.050 | VF (%) | 0.610 (0.321-0.899) | 0.147 |
| WHR (cm) | 0.604 (0.508-0.700) | 0.049 | WHR (cm) | 0.594 (0.301-0.887) | 0.149 |
| Women >40 years old |  |  |  |  |  |
| VF (%) | 0.581 (0.523-0.639) | 0.029 | WHR (cm) | 0.645 (0.577-0.713) | 0.034 |
| WC (cm) | 0.569 (0.511-0.627) | 0.029 | WHtR (cm) | 0.636 (0.570-0.702) | 0.033 |
| WHtR (cm) | 0.569 (0.511-0.627) | 0.029 | VF (%) | 0.633 (0.566-0.700) | 0.034 |
| WHR (cm) | 0.559 (0.501-0.617) | 0.029 | WC (cm) | 0.605 (0.539-0.671) | 0.033 |
| BMI (kg/m^2^) | 0.534 (0.474-0.593) | 0.030 | BMI (kg/m^2^) | 0.511 (0.440-0.582) | 0.036 |
| BF (%) | 0.513 (0.453-0.573) | 0.030 | BF (%) | 0.510 (0.438-0.581) | 0.036 |
| BF, body fat; BMI, body mass index; VF, visceral fat; WC, waist circumference; WHR, waist to hip ratio; WHtR, waist to height ratio.  Data shown Receiver Operating Characteristics Distribution of the areas under curves considering the criterion variable the prehypertension (SBP: 120-139 mmHg/DBP: 80-89 mmHg) and hypertension (SBP: ≥140 mmHg/DBP: ≥90 mmHg). | | | | | |
